# Global and Multi-National Prevalence of Fungal Diseases—Estimate Precision

**DOI:** 10.3390/jof3040057

**Published:** 2017-10-18

**Authors:** Felix Bongomin, Sara Gago, Rita O. Oladele, David W. Denning

**Affiliations:** 1 The National Aspergillosis Center, Education and Research Centre, Wythenshawe Hospital, Manchester University NHS Foundation Trust, Manchester M23 9LT, UK; felix.ayoli9@gmail.com; 2 Division of Infection, Immunity and Respiratory Medicine, Faculty of Biology, Medicine and Health, The University of Manchester, Manchester M13 9PL, UK; sara.gago-2@manchester.ac.uk (S.G.); rita.oladele@postgrad.manchester.ac.uk (R.O.O.); 3 Global Action Fund for Fungal Infections, 1211 Geneva 1, Switzerland; 4 Manchester Fungal Infection Group, Core Technology Facility, The University of Manchester, Manchester M13 9PL, UK

**Keywords:** global prevalence, fungal diseases, estimate precision

## Abstract

Fungal diseases kill more than 1.5 million and affect over a billion people. However, they are still a neglected topic by public health authorities even though most deaths from fungal diseases are avoidable. Serious fungal infections occur as a consequence of other health problems including asthma, AIDS, cancer, organ transplantation and corticosteroid therapies. Early accurate diagnosis allows prompt antifungal therapy; however this is often delayed or unavailable leading to death, serious chronic illness or blindness. Recent global estimates have found 3,000,000 cases of chronic pulmonary aspergillosis, ~223,100 cases of cryptococcal meningitis complicating HIV/AIDS, ~700,000 cases of invasive candidiasis, ~500,000 cases of *Pneumocystis jirovecii* pneumonia, ~250,000 cases of invasive aspergillosis, ~100,000 cases of disseminated histoplasmosis, over 10,000,000 cases of fungal asthma and ~1,000,000 cases of fungal keratitis occur annually. Since 2013, the Leading International Fungal Education (LIFE) portal has facilitated the estimation of the burden of serious fungal infections country by country for over 5.7 billion people (>80% of the world’s population). These studies have shown differences in the global burden between countries, within regions of the same country and between at risk populations. Here we interrogate the accuracy of these fungal infection burden estimates in the 43 published papers within the LIFE initiative.

## 1. Introduction

Nearly a billion people are estimated to have skin, nail and hair fungal infections, many 10’s of millions mucosal candidiasis and more than 150 million people have serious fungal diseases, which have a major impact on their lives or are fatal. However, severity ranges from asymptomatic-mild mucocutaneous infections to potentially life-threatening systemic infections. Moreover, mortality associated with fungal disease at >1.6 million is similar to that of tuberculosis and >3-fold more than malaria. Socio-economic, geo-ecological characteristics and the increasing number of at-risk populations are the main determinants of variations on incidence and prevalence of fungal disease across the world. HIV/AIDS pandemic, tuberculosis, chronic obstructive pulmonary disease (COPD), asthma and the increasing incidence of cancers are the major drivers of fungal infections in both developed and developing countries globally [1,2,3,4,5,6].

Recent global estimates found 3,000,000 cases of chronic pulmonary aspergillosis, ~223,100 cases of cryptococcal meningitis complicating HIV/AIDs, ~700,000 cases of invasive candidiasis, ~500,000 cases of *Pneumocystis jirovecii* pneumonia, ~250,000 cases of invasive aspergillosis, ~100,000 cases of disseminated histoplasmosis, over 10,000,000 cases of fungal asthma and ~1,000,000 cases of fungal keratitis occur annually (Table 1) [1,7,8,9]. Here we address these and estimates for the many countries that contribute to these global figures.

Although the epidemiology of fungal diseases has greatly changed over the past few decades, *Aspergillus*, *Candida*, *Cryptococcus* species, *Pneumocystis jirovecii,* endemic dimorphic fungi such as *Histoplasma capsulatum* and Mucormycetes remain the main fungal pathogens responsible for the majority cases of serious fungal disease. *Candida albicans* is the main agent responsible for mucosal disease, *Aspergillus fumigatus* for most allergic fungal disease and *Trichophyton* spp., especially *T. rubrum*, for skin infections.

**Figure 1:**
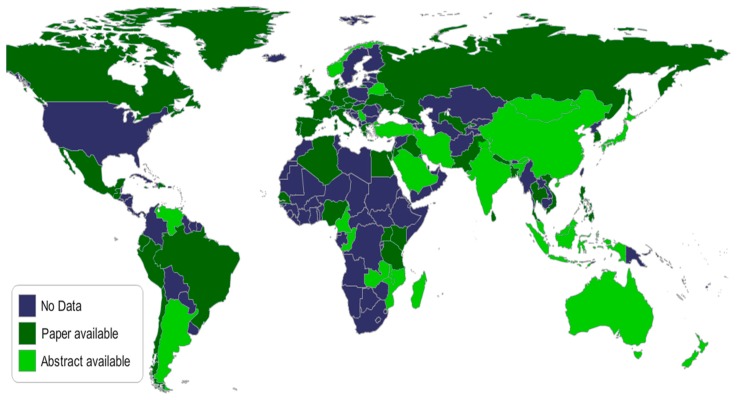
A map showing completed country estimates of fungal diseases by August 2017.

In the last four years, the Leading International Fungal Education (LIFE) portal has facilitated the estimation of the burden of serious fungal infections country by country for over 5.7 billion people (>80% of the world’s population) (Figure 1) [14]. These studies have shown differences in the global burden between countries, within regions of the same country and between at risk populations.

Global estimates, with individual country breakdowns, have been estimated for chronic pulmonary aspergillosis after pulmonary tuberculosis and complicating sarcoidosis, of allergic bronchopulmonary aspergillosis complicating asthma and cystic fibrosis, *Aspergillus* bronchitis complicating cystic fibrosis and mostly recently a revised estimate of cryptococcal meningitis in AIDS and recurrent vulvovaginal candidiasis [8,11,15,16,17,18]. However, a precise estimate of global prevalence and incidence for each fungal infection remains unknown and, data are scanty most countries, especially in the developing word.

Knowledge about the global incidence of fungal diseases has been impaired by lack of regular national surveillance systems, no obligatory reporting of fungal diseases, poor clinician suspicion outside specialised units, poor diagnostic test performance (especially for culture) and few well-designed published studies. Some fungal diseases are only recently recognised [2,9,19].

Over 80% of patients could be saved from dying with universal availability of fungal diagnostics and potent antifungals agents, based on well documented treatment response rates. However, the early recognition and management of serious fungal infections is always a challenge, but especially in resource-limited settings as many conventional diagnostics tests are slow, antifungal treatment can be expensive and/or toxic and is not equally available in all countries. Other factors impinging on better outcomes include patient compliance with long-term treatment, drug-drug interactions, limited clinical experience of excellent care in many settings and co-morbidities reducing the potential for survival and cure [20].

GAFFI has put together a list of priority fungal diseases that are of public health importance, and amenable to improved diagnosis and better treatment outcomes. These include cryptococcal meningitis, *Pneumocystis* pneumonia, disseminated histoplasmosis, chronic pulmonary aspergillosis, and fungal keratitis [20]. Modelling with existing outcome data shows that mortality associated with these priority fungal diseases concurrently with the Joint United Nations Program on HIV/AIDS (UNAIDS) 90-90-90 campaign could save over 1.6 million lives of persons living with HIV globally over the next five years [2]. The WHO has recently accepted Mycetoma and Chromoblastomycosis as Neglected Tropical Diseases [21,22,23].

In this article, we overview how estimates of serious fungal diseases were derived and the strengths and weaknesses of these methods from the 43 published reports (not abstracts) of >2000 million people (29% of the world population), with examples.

## 2. Estimate precision

### 2.1. Candidaemia

#### 2.1.1. 5142 Cases of Invasive Candidiasis in the UK

In 2016, Pegorie et al. [19] estimated the annual incidence of invasive candidiasis at 5142 cases in the, U.K. There were 1700 voluntary laboratory reports of candidaemia in 2013 in England, Wales and Northern Ireland. These were assumed to represent about 38% cases of proven or probable invasive candidiasis tested by blood culture techniques, based on a pooled culture positivity rate in patients with proven or probable invasive candidiasis of 0.38 (95% Confidence Interval: 0.29–0.46) [24,25,26]. The resulting estimate for the total number of cases in England, Wales and Northern Ireland in 2013 was thus 4473. Scotland had a rate of candidaemia of 4.8 cases per 100,000 population per year shortly after the millennium, 254 bloodstream and 669 invasive *Candida* cases annually [27]. The total estimate of invasive candidiasis burden for the UK was therefore: 5142 (4473 + 669).

#### 2.1.2. Discussion

The incidence of nosocomial candidiasis has increased over the past few decades [28,29,30,31,32]. Moreover, *Candida* bloodstream infection incidence is bimodal, with older people and premature babies having the highest risk. Candidaemia is the most common form of invasive candidiasis associated with an unacceptably high mortality rates in excess of 40% even with the introduction of newer antifungal agents [30,32,33]. The reasons for the increasing incidence of invasive candidiasis are thought to be due to the use of broad-spectrum antibiotics and immunosuppressant agents, higher survival of premature infants, prolonged hospital and intensive care unit (ICU) stay, diabetes, nosocomial bacterial infection, recent surgery, notably major intra-abdominal procedures, pancreatitis, mechanical ventilation, total parenteral nutrition, use of medical devices such as central venous catheter and shunts, increased transplantation and more immunocompromising medical conditions such as malignancies [29,30,33,34,35,36,37,38,39,40]. About 10% of cases occur in the community, but almost always in those with one or more of the above diagnoses. This multiplicity of underlying disorders and concentration of such patients in referral centres complicates burden estimations.

*Candida albicans* is the main etiologic *Candida* species associated with nosocomial invasive candidiasis globally [41]. However, there has been a worrying increase in the number of non-*albicans Candida* species such as *C. glabrata*, *C. parapsilosis*, *C. tropicalis*, *C. krusei* and lastly *C. auris*. These species are more likely to be antifungal resistant and have the potential to cause outbreaks [42]. In particular, resistance to fluconazole is common [30,43,44] which is important as it is the most commonly used antifungal agent for prophylaxis and treatment of *Candida* infections in many parts of the world [45].

For the 43 published papers estimating the burden of fungal infections in each country, 39 of them reported data about candidaemia. The total burden of candidaemia is 159,253 cases across these 39 countries with available data. Sixty percent of the cases were reported in the ICU followed by cancer and transplant units (13%). The highest prevalence of candidaemia was reported in Pakistan (38,795 cases, 21 cases per 100,000) followed by Brazil (28,991 cases, 14.9 cases per 100,000) and Russia (11,840 cases, 8.29 cases per 100,000). The lowest incidence figures were reported in Jamaica (136 cases, 5 per 100,000), Austria (206 cases, 2.1 per 100,000) and Portugal (231 cases, 2.2 per 100,000). The incidences in the three most populous countries, China, India and US have not been published using this methodology. Comparisons of the incidence across countries is difficult as recent local epidemiological data was only available for 20 of the countries and the others used an incidence of 5 per 100,000 cases for their estimations [46]. Many low and low middle income countries do not have blood culture systems in the any or the majority of hospitals. Moreover, the lack of local epidemiological data in all countries together with differences in the quality of the data—not all institutions within the same country report their *Candida* infections—indicate that the candidaemia global burden is underestimated.

Differences across continents in candidaemia are quite important. Fifty percent of the global cases of candidaemia were reported in Asia (*n* = 10 countries, 78,778 cases) followed by the Americas (*n* = 10 countries, 40,613 cases; 33,962 in Central and South America), Europe (*n* = 1 country, 420,549 cases) and Africa (*n* = 5 countries, 19,602 cases). The incidence of candidaemia was higher in middle income countries and this can be linked to compromised healthcare systems, deficiency in resources, poor infection control implementation, unavailability of diagnostic tests allowing excess empirical therapy, impaired knowledge about fungal infections or misuse of antibiotics without stewardship programs [47].

For 29 countries, the prevalence of *Candida* peritonitis (intra-abdominal candidiasis) was also estimated (Table 2). *Candida* peritonitis global burden in these countries is 17,640 cases with an average incidence of 1.15 cases per 100,000. Most of the studies have estimated the burden of *Candida* peritonitis as 50% of the total cases of invasive candidiasis occurred in the ICU unit. The highest *Candida* peritonitis burdens were reported in Mexico (5596; 4.98/100,000) followed by Germany (3700; 4.6 per 100,000), Nigeria (2321; 1.5 per 100,000) and Spain (668; 1.42 per 100,000). The high morbidity and mortality of *Candida* peritonitis in developed countries has been associated with the age of patients, the use of antibiotics and corticosteroid treatment [48,49,50,51].

### 2.2. Invasive Aspergillosis

#### 2.2.1. 294 Annual Cases of Invasive Aspergillosis in Denmark

Mortensen et al. [58] estimated the burden of invasive aspergillosis (IA) and other fungal diseases in Denmark. The estimation of the burden of IA in allogenic haematopoietic stem cell transplantation (HSCT) and solid organ transplantation was based on data published by Herbrecht et al. [94] and Iversen et al. [95]. They assumed proven and probable IA in the transplant (Tx) population happened in 4/41 HSCT cases (10%), 2/214 renal Tx cases (1%), 2/30 lung Tx cases (6%), 2/26 cases of heart Tx (6%) and 2/48 cases of liver Tx (4%) giving a total of 12 annual cases of IA in transplant populations.

A total of 2,152 leukaemia cases were registered in Denmark in 2012. To estimate the burden of IA in haematological disease they took into account the prevalence of IA in patients with haematological malignance published by Pagano et al. in Italy [96]. The number of IA cases by haematological disease was distributed as follows, 13/183 in acute myeloid leukaemia (7%), 2/65 in acute lymphoblastic leukaemia (3.75%), 2/67 cases in chronic myeloid leukaemia (2.35%), 1/274 IA cases in chronic lymphatic leukaemia (0.45%), 1/314 IA cases in multiple myeloma (0.25%) and 8/1046 cases in non-Hodgkin lymphoma (0.78%) giving a total of 27 cases of IA in patients with haematological disease.

Finally, for 19,693 cases of COPD admitted to hospital, some to intensive care, [4], 1.3% were predicted to develop IA (from Spain) giving a burden of 255 IA cases in this setting. An annual incidence of 294 cases of invasive aspergillosis was estimated in Denmark.

#### 2.2.2. Discussion

Invasive aspergillosis is a severe and aggressive fungal disease that occurs in profoundly immunocompromised hosts [97]. In these patients, with impaired immune function, *Aspergillus* growth in the lung leads to tissue destruction, angio-invasion, a septic state in its final phase and sometimes haemoptysis [98]. The highest risk populations for invasive aspergillosis comprise chronic granulomatous disease, prolonged neutropenia, HSCT and heart, lung and pancreas organ transplantation [99,100]. Other lower risk patients include those with COPD [4], high dose corticosteroid treatment [101], lung cancer [102], liver cirrhosis [103,104], renal and liver transplantation [105], diabetes mellitus [106] and sepsis, especially in the ICU setting [107]. The global incidence of invasive aspergillosis was previously reported to be 200,000 cases with an associated mortality ranging from 30–80% [108], recently updated to >300,000 cases [20].

Probably the total and variability in burden of IA is driven by one main factor (COPD), as other contributions will be small or relatively constant (i.e., the rate of acute myeloid leukaemia). Two studies have documented the rate of IA detected by culture in patients admitted to hospital with IA: Guinea et al. [4] in Spain and Xu et al. [109] in southern China. In the first the rate of IA was 1.3% in the final year (2007) of their study and 3.9% in the second study. Another small study in intubated patients with COPD and a pulmonary infiltrate in Brazil, found 4.2% with a positive culture and 21.3% with high galactomannan levels in respiratory fluids [110]. Assuming that IA is restricted to those ill enough to be admitted to hospital, the denominator for IA cases varies from as low in ~25 to 375 per 100,000 people over 40 years and older in 2011 in Japan and Hungary respectively among the Organisation for Economic Co-operation and Development (OECD) countries [111]. In Europe, there are 1,100,000 COPD admissions annually, possibly contributing 14,300–42,900 IA cases [112]. In China, there are 11,858,100 admissions to hospital per year, probably contributing 154,155–462,466 IA cases in COPD [113]. Some estimates have also included IA complicating lung cancer, estimated from one large study in China at 2.6% [102]. If this rate pertains to the global population of lung cancer (1,824,700) [114] then 47,500 cases of IA are anticipated. Given these data, the global estimate of >300,000 cases of IA is almost certainly a significant underestimate, but more data are required.

Here, we have reviewed the incidence and prevalence of invasive aspergillosis in 40 countries accounting for a global population higher than 2000 million people (29% of the world population) (Table 3). 81,927 cases of invasive aspergillosis have been observed with the maximum number of cases in Vietnam (14,523 cases a year) [115] and the minimum number of cases in Trinidad and Tobago (8 cases a year) [79]. Average incidence was 4.10 cases/per 100,000 with highest and lowest values in Vietnam (16 cases per 100,000) and Tanzania (0.05 cases per 100,000) [78] respectively. For Jamaica and Senegal no estimate of the burden of invasive aspergillosis was possible. The lack of data on COPD admission to hospital is a limiting factor, although published for several countries [116,117]. In no country were estimates of IA complicating AIDS included, although found in ~4% of autopsies in developed countries [118], in the era prior to, A.R.T.; but still present [119]. A global estimate has been made of IA in AIDS of >45,000 cases [20].

**Figure 2:**
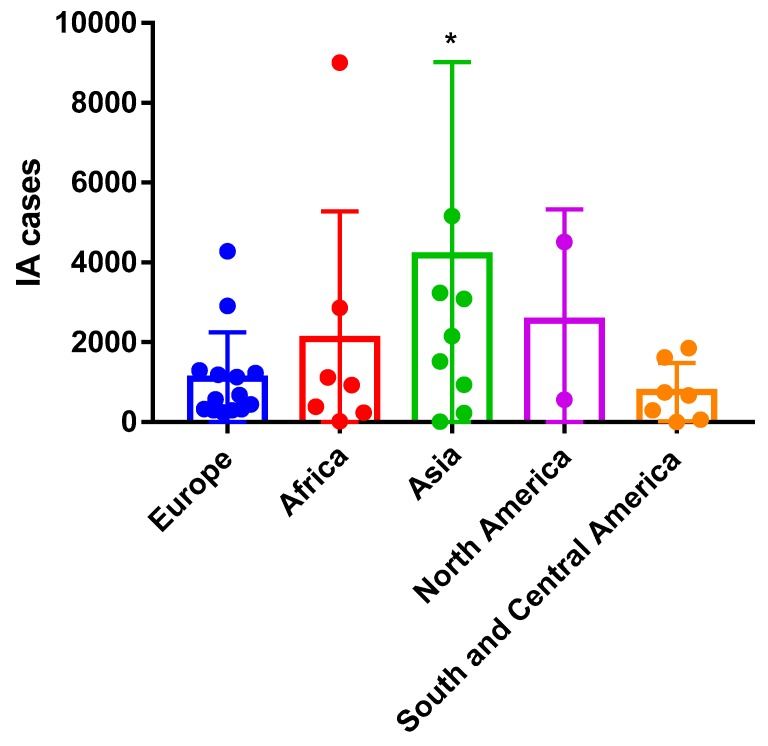
Prevalence of invasive aspergillosis in a continental level. * *p* < 0.05.

We further explore differences in the incidence and prevalence of invasive aspergillosis in a continental level. We found 50% of the estimated cases in Asian countries (41,813) (not including India or China) followed by Europe and Asia (Figure 2). However, there are some limitations in this data due to differences in size sample between countries and the diagnostics capabilities.

Distribution of the number of cases of invasive aspergillosis in different medical units including HIV/AIDS, respiratory, cancer and transplantation and ICU was also analysed in 39/43 countries. None of the cases were reported in HIV/AIDS. No differences in the total number of cases across units were reported.

### 2.3. Pneumocystis jirovecii Pneumonia

#### 2.3.1. Over 700 Cases of *Pneumocystis jirovecii* Pneumonia Per Year in Guatemala

*Pneumocystis jirovecii* pneumonia (PCP) mainly occurs in the context of HIV-associated immunosuppression. Medina et al. [123] estimated the burden of fungal diseases in Guatemala and concluded that about 271,577 cases (1.7% of the population), 722 of these cases were because of PCP. In 2015, the estimated number of adult people living with HIV in Guatemala was 53,000, of whom 30% (15,900) were on ARV therapy [124]. An assumption was made that 64% (0.64 × (53,000 − 15,900) = 23,744) of adults, who are not on, A.R.V.; with CD4 count <200/μL cells are immediately susceptible to fungal disease. A preliminary study from an urban clinic in Guatemala had shown that 4.7% of cases of PCP are found at the time of HIV diagnosis [125], thus, 1,116 (4.7% of 23,744) cases of PCP. Adjusted for deaths and the fact that most cases of PCP in Guatemala are clinically diagnosed based on symptoms and differential diagnosis, without laboratory confirmation, a figure of 722 cases per year was reached.

#### 2.3.2. Discussion

*Pneumocystis jirovecii* pneumonia is emerging as a leading cause of infection in HIV/AIDS patients [1]. Global prevalence is thought to be higher than 400,000 annually cases worldwide [3]. Mortality of PCP ranges from 10–30% and can be even higher if the diagnosis is delayed [6]. Although the incidence has been reduced after the implementation of the HAART therapy, it is still high in HIV misdiagnosed patients, in those who do not have access to HAART and in those who stop HAART treatment [126,127]. In addition, the increasing use of immunosuppressant drugs, their use in high doses or their combination has increased numbers of PCP cases in patients with solid tumours, haematological malignancies, rheumatic diseases or organ transplant recipients [128,129]. The main challenge in the management of patients with PCP remains achieving an early diagnosis. Definitive diagnosis is based on the identification of *P. jirovecii* in respiratory samples [130]. As this microorganism cannot be cultured in laboratory conditions, direct examination and immunofluorescence are used as reference methods with sensitivity rates higher than 90% [128,131], or PCR methods in developed countries [132]. However, all these methods require trained personnel that are not available in all settings.

The burden of PCP has been included in 40 published papers within the LIFE programme. A total of 133,487 cases have been estimated across the countries included in the study. The highest burdens have been estimated in Nigeria (74,595), Kenya (17,000) and Tanzania (9600). On the other hand, the lowest PCP burdens have been reported in Denmark (2), Hungary (5), Qatar (15) and Israel (26). The global incidence was estimated as 5.79 (SD ± 10.96) cases per 100,000. The highest and lowest incidence was reported in Nigeria (48.3) and Bangladesh (0.04) cases per 100,000 respectively. Seventy-seven percent of the cases (102,955) were reported in Africa, followed by America (10%) Europe (7%) and Asia (6%). Differences in the estimations across countries can be associated to differences in the HIV prevalence in the different countries and the accessibility to highly active antiretroviral therapy with rates ranging from 2–60% in AIDS populations (Table 4).

### 2.4. Chronic Pulmonary Aspergillosis (CPA)

#### 2.4.1. About 120,754 Cases of CPA in Nigeria

Oladele et al. [62] estimated very high rate of CPA among Nigerians at 78 cases per 100,000 individuals. This estimate was based on previously published report on CPA as a sequel of TB by Denning et al. [15]. About 78,032 cases of pulmonary TB were reported in Nigeria in 2010, most in HIV negative people and based on pulmonary cavity frequency and *Aspergillus*-specific IgG serology, 19,000 new cases of CPA were expected annually with a 5-year period prevalence of 60,377 cases. This assumes a 15% annual mortality. Prior TB is perhaps the most common underlying condition in, C.P.A.; post-tuberculous cases probably represent 50% (range 20–85%) [134] of the total CPA caseload, therefore the estimated cases of CPA in Nigeria was (60,377 × 2) = 120,754.

#### 2.4.2. Discussion

CPA is an uncommon or rare pulmonary disease complicating other respiratory disorders such as tuberculosis, COPD or sarcoidosis. The term CPA includes simple aspergilloma, chronic cavitary pulmonary aspergillosis and chronic fibrosing pulmonary aspergillosis. CPA is thought to affect about 3 million people worldwide and, if untreated 80% of patients with CPA will die within 5 years [97]. In a cohort of 387 CPA patients referred to the UK’s National Aspergillosis Centre from 1992 to June 2012, survival was 86%, 62% and 47% at 1, 5 and 10 years, respectively [135].

**Figure 3:**
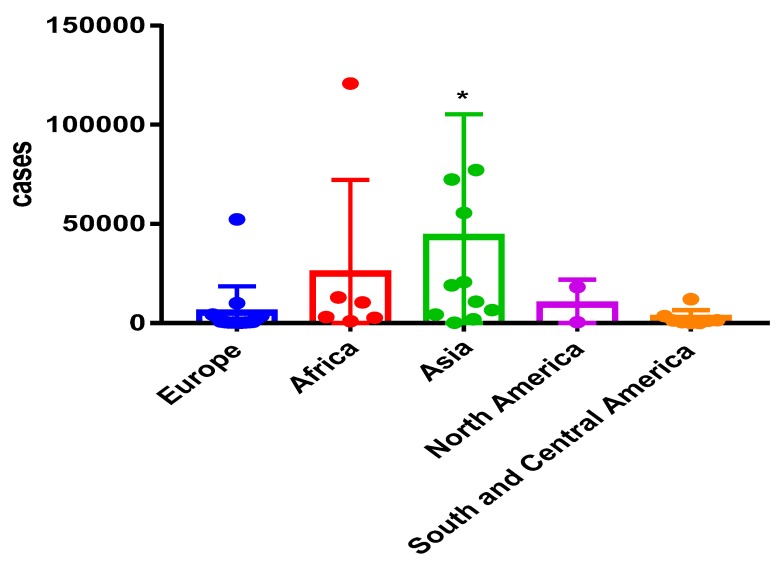
CPA prevalence across continents. * *p* < 0.05.

The annual incidence of CPA after TB and 5 year period prevalence of CPA have been reported so far in 43 countries, accounting for 45% of the global population, including India. The highest burden of CPA has been reported in India (209,147) followed by Nigeria (120,753), Philippines (77,172), Pakistan (72,438) and Vietnam (55,509); the lowest burdens were reported in Jamaica (82), Trinidad and Tobago (110), Qatar (176) and Ireland (196). Burdens in Asia were significantly higher to any other country (*p* < 0.01) (Figure 3).

The incidence for all the countries included in the review was 22 cases per 100,000 (14.2–30.59 95% CI) with the lowest incidence in Canada (1.38 cases per 100,000), Algeria (2.2 cases per 100,000), Israel (2.5 cases per 100,000) and Germany (2.9 cases per 100,000); the highest incidence were estimated in Russia (126.9 cases per 100,000) followed by Philippines and Nigeria (78 cases per 100,000), Pakistan (70 cases per 100,000) and Vietnam (61 cases per 100,000) (Table 5).

### 2.5. Allergic Bronchopulmonary Aspergillosis (ABPA) & Severe Asthma with Fungal Sensitisation (SAFS)

#### 2.5.1. Up to 28,447 Patients with ABPA and 37,491 with SAFS in Ukraine

From the estimates by Osmanov et al. [80], 28,447 patients were estimated to have ABPA and 37,491 with SAFS in Ukraine. The major assumptions made were based on previous studies of SAFS and ABPA; Severe asthma is thought to affect about 10% of adults and in other countries, 33–70% are sensitised to fungi [137] and that 2.77–2.90% of population have asthma [136]. A total of ~1,136,092 adults are estimated to have asthma in Ukraine. Assuming 2.5% of asthmatics have ABPA Osmanov estimated that there are 28,447 [2.5% of 1,136,092] (62 per 100,000) patients with ABPA and 37,491 [3.33% of 1,136,092] (37.5 per 100,000) with severe asthma with fungal sensitisation (SAFS) (using a 33% sensitisation rate). In this paper, no numerical allowance was taken for the likely duplication of some cases of ABPA and SAFS, although this was done in a recent UK estimate [19].

#### 2.5.2. Discussion

Allergic bronchopulmonary aspergillosis (ABPA) is usually a progressive allergic lung disease to *Aspergillus* antigens [97]. ABPA is a common complication of asthma or cystic fibrosis. Hypersensitivity to *Aspergillus fumigatus* is reflected by a positive skin test or high levels of *Aspergillus* specific IgE. Clinical symptoms include poorly controlled asthma, haemoptysis, fever and expectoration of mucus plugs [138,139]. A related term allergic bronchopulmonary mycosis (ABPM) is used when the causative fungus is not *Aspergillus* spp. Both ABPA and ABPM often manifest as poorly-controlled asthma or recurrent infection because of bronchiectasis. They may develop progressive lung damage, respiratory failure and eventually death [140]. The mean ABPA prevalence rate of 2.5% in adult asthmatics reflects studies done in new asthma referrals to a specialist in South Africa, New Zealand, Iran, Ireland, Saudi Arabia and China, translating to over 4.8 million cases of ABPA in adults [137]. There no published population studies on this condition.

Asthma is a chronic respiratory disease associated with significant increase in the years of life lost due to premature mortality, the age-standardised mortality rates for asthma dropped from 8.8/100,000 in 2005 to 6.1/100,000 by 2015 [141]. The global prevalence rates of doctor diagnosed asthma, clinical asthma and wheezing in adults is estimated at 4.3%, 4.5%, and 8.6% respectively among individuals aged 18 to 45 years [142]. The lifetime prevalence of asthma increases with age, a Scottish study reported asthma point prevalence of 20% in boys and 12% in girls under 16 years with a corresponding lifetime prevalence of 29% and 20% respectively [143].

ABPA burden has been estimated to be a total of 3,550,157 (SD ± 213,051) cases in 43 countries (Table 6). Prevalence primarily reflects the adult asthma prevalence per country, as ABPA in children is rare, although reported [144]. The highest prevalence rate has been reported in India (1,380,000 cases (range 120,000–6,090,000). High quality large ABPA prevalence studies have not been done in India, but the proportion of asthmatics with ABPA appears to be higher than any other country [136]. High prevalence has also been estimated in Brazil (390,486 cases), Israel (331,876 cases) and United Kingdom (235,070) and the lowest burdens estimated in Uzbekistan (879 cases), Qatar (1126), Trinidad and Tobago (3491) and Czech Republic (4739). ABPA average incidence was 116 cases/100,000 with the highest and lowest rates reported in Israel (393 cases per 100,000) and Vietnam (23 cases per 100,000). A negative correlation between ABPA and GDP was found (*r* = −0.22, *p* = 0.04).

**Figure 4:**
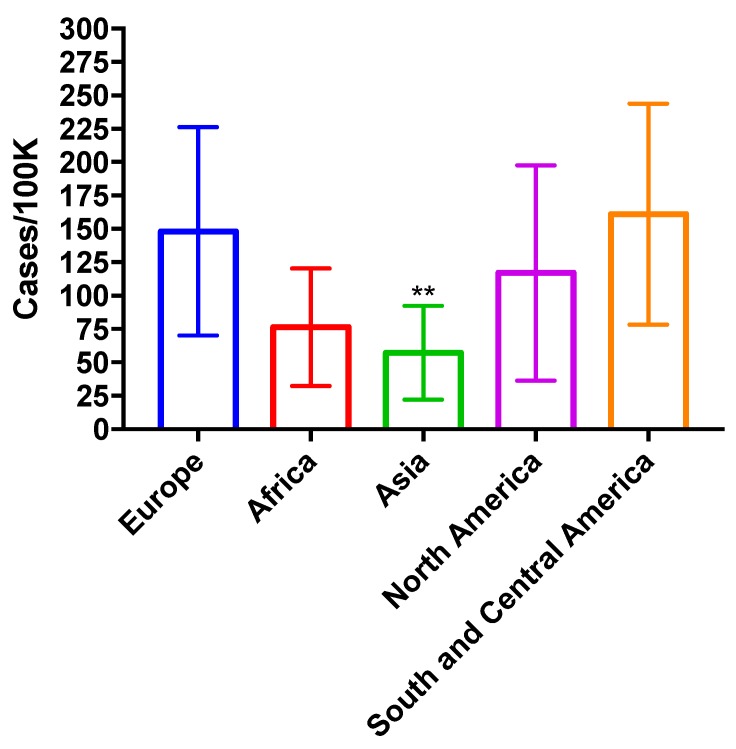
ABPA prevalence across countries. ** *p* < 0.01.

No differences in ABPA incidence across countries was found, however, incidence in Asia was significantly lower than in other countries with the exception of India and Pakistan (Figure 4).

Asthmatic patients with severe disease (British Thoracic Society step 4 or worse ) who have evidence of sensitized to one or more fungi, by skin prick test or RAST test but do not meet the diagnostic criteria for ABPA (total IgE < 1000 IU/mL) are classified as patients with Severe Asthma with Fungal Sensitization (SAFS) [145]. Fungal sensitization in asthma, mainly due to *Aspergillus* spp. leads to increased severity, more exacerbations and a higher mortality [146,147]. It is estimated that 3% of adult asthmatics will have SAFS and this might account for more than 6.5 million cases globally [145]. Although the prevalence of SAFS is uncertain as this disease has been recently described, it was conservatively estimated that 30% of patients with severe asthma have or develop SAFS. Patients with SAFS probably constitute a significant proportion (~50% estimated) of the nearly 0.5 million asthma related deaths worldwide [148,149].

**Figure 5:**
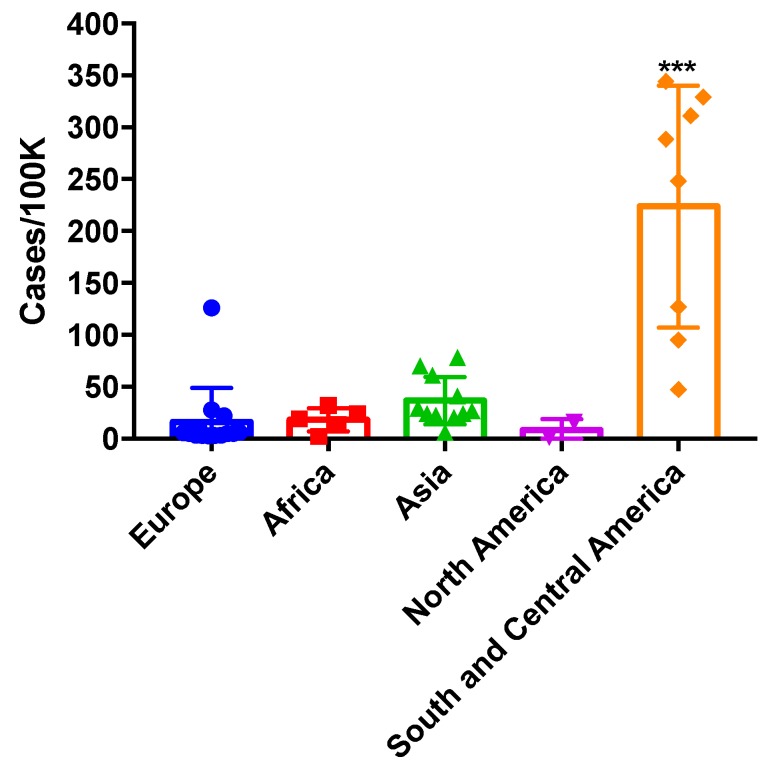
SAFS prevalence across continents. *** *p* < 0.001.

The burden of SAFS has been estimated to 4,125,428 (±174,033) cases in 43 countries (Table 7). The highest burdens have been reported in India (960,000 cases), Brazil (599,283 cases) and United Kingdom (413,724 cases), the lowest prevalence rates have been reported in Uzbekistan (1147 cases), Qatar (1486 cases) and Trinidad and Tobago (4608 cases). Average incidence is 154 cases per 100,000 (SD ± 654) with the highest incidence rates reported in United Kingdom (654 per 100,000) and lowest in Vietnam (34 cases per 100,000) and Uzbekistan (3.7 per 100,000) respectively. The very low prevalence in Uzbekistan. Interestingly, SAFS incidence in South and Central America was significantly higher (*p* < 0.0001) than in the other regions analysed (Figure 5).

### 2.6. Fungal Keratitis

#### 2.6.1. 11,638 Cases of Fungal Keratitis in Mexico

Fungal keratitis is an implantation infection, related to injury or contact lens wear. As such there are no underlying diseases from which to estimate rates. Almost no data are published on fungal keratitis in Central or South America. Corzo-Léon et al. [76] estimated the burden of serious fungal infections in Mexico based on systematic literature search of epidemiological studies reporting these diseases. The reported burden of fungal keratitis was 11,638 (rate 10.4 per 100,000), making fungal keratitis the 4th of the top 10 of serious fungal diseases in Mexico after rVVC, ABPA, and CPA. The burden of infectious (all-cause, including fungal) keratitis in Mexico was estimated based on the prevalence of reported in China (0.148%) [150]. As the Mexican population in 2010 was 112,336,538, so 166,258 (0.148%) cases of microbial keratitis are likely, assuming individuals of all ages and genders are at risk of acquiring this disease. The previously reported proportion of fungal keratitis amongst all causes of microbial estimated from locally available Mexican data was 7% (6.1–7.9) [151], which is a low proportion for tropical and sub-tropical countries. Thus the burden of fungal keratitis was estimated at (7% of 166, 258) = 11,638 cases.

#### 2.6.2. Discussion

Fungal keratitis (also known as mycotic keratitis or keratomycosis) refers to corneal infection caused by any of the fungal genus usually *Aspergillus*, *Fusarium*, *Candida*, *Phoma*, *Basidiomycetes*, or Mucorales. Fungal keratitis is more common in tropical countries where it constitutes between 20 and 60% of all culture-positive cases of corneal infections primarily following ocular trauma [152]. Mycotic keratitis remains a serious cause of corneal opacification and vision loss [153], with an estimated global burden of about 1–1.2 million cases annually [1]. In a recent 11-year prospective study evaluating visual outcomes of 1130 patients diagnosed with fungal keratitis from Larkana, Pakistan, 126 (11%) patients lost their eyeballs and over 50% of the remainder effectively lost their sight [154]. The incidence is lower in developed countries, for example a recent study from the Republic of Ireland estimated the minimum incidence of fungal keratitis at 1.53 cases per million population per year [155]. However, a large retrospective survey conducted in the U.K between 2004 and 2015 showed a rise in the annual incidence of fungal keratitis as percentage of all cases of microbial keratitis from 5.2% in 2004 to 9.5% in 2015 [156].

Fungal keratitis is not rare in tropical countries; however, the burden has been estimated in very few countries (Table 8). Prior estimates of incidence have been published, but are decades old in many cases [157,158,159]. Fungal keratitis is often under-suspected hence underdiagnosed and it is fairly difficult to diagnose; sample collection (corneal scrapings) requires eye specialist doctors/nurses with specialised equipment (slit lamp). There is no point-of-care test for fungal keratitis. Of the published burdens, very high incidence rates have been reported in countries such as Nepal, Pakistan, Thailand, Egypt, and Mexico. The Philippines, Korea, Denmark, and Germany are among the countries with the lowest incidences of fungal keratitis, although the rate in the Philippines could reflect low self-referral and diagnostic rates. Local epidemiological studies are required to improve these estimates and generate first estimates for many countries, especially Africa and Central and South America.

### 2.7. Recurrent Vulvovaginal Candidiasis

#### 2.7.1. About 6% (443,237 of 7,380,000) of Nepalese Women Suffer from Recurrent Vulvovaginal Candidiasis

Khwakhali et al. [121] estimated burden of serious fungal disease in Nepal. By 2014, Nepal had 7,380,000 women aged between 15 and 49 years. The number of women probably suffering from recurrent vulvovaginal candidiasis (rVVC) was estimated by assuming that rVVC affects 6% of adult women aged between 14 and 55 years and ‘recurrent’ defined as at least four episodes per year [163]. From this assumption, 443,237 (6% of 7,380,000) cases of rVVC were estimated.

#### 2.7.2. Discussion

Vulvovaginal candidiasis affects 70–75% of women at least once during their lives, mainly during childbearing age [164,165]. Recurrent VVC (defined as four or more episodes every year) is a common cause of significant morbidity in women in all strata of society affecting millions (5–9%) of adult women. It occurs worldwide with a maximum prevalence between 25 and 34 years of age [163,166,167,168]. The global prevalence of rVVC is estimated at about 134,000,000 cases [18]. The pathogenesis of rVVC is poorly understood; episodes of VVC may be triggered by antibiotic use, sexual activity, high carbohydrate diet, local or systemic corticosteroids, oestrogen (as in hormone replacement therapy); usually no predisposing factor can be identified [164].

In most of the country burden estimates, a uniform rate of 6% was used to estimate the national burden of rVVC and episodes occurring after the menopause were ignored. Data supporting this proportion of women affected are missing from Central and South America, Africa, Middle East, Asia and Australasia, although the problem of rVVC is seen in all populations.

### 2.8. Tinea Capitis

#### 2.8.1. Over 15,500,000 Cases of Tinea Capitis among Nigerian Children

Oladele and Denning estimated 17,983,517 cases of serious fungal diseases in Nigeria in 2014, 15,581,400 (86.6%) of which were attributed to tinea capitis [62]. Fifty percent of the 155+ million Nigerian populations are children (~77,500,000). Local estimates of tinea capitis exceed 20% of school age children [169,170,171], suggesting that over 15,500,000 (20% of 77,500,000) children have tinea capitis [62]. One assumption was also made that no adults had tinea capitis.

#### 2.8.2. Discussion

Tinea capitis is a common superficial infection of the scalp hair caused by dermatophyte fungi; occurring predominantly in children [172,173]. Its clinical manifestations range from mild scaling with little hair loss to large inflammatory and pustular plaques with extensive alopecia [173]. Tinea capitis can be caused by a variety of anthropophilic or zoophilic *Trichophyton* or *Microsporum* species. Tinea capitis was previously prevalent in a number of industrialised countries in the early twentieth century, it was brought under effective control following the introduction of griseofulvin and concerted public health interventions [173,174]. The species of dermatophyte implicated in tinea capitis infections vary from geographic region to region. In turn, this pattern also changes with time, particularly as new organisms are introduced by migration or immigration [173]. A recent review revealed a change in the last 1–2 decades, the spread of *Trichophyton tonsurans* as the dominant agent of tinea capitis, in the Americas, UK, Europe and Africa [173]. There has also been reports of *T. tonsurans* in Japan which has been associated with infection in older children and wrestlers [173]. *T. violaceum* is the most common human etiological agent in Eastern and Southern parts of Africa, with prevalence ranging from 56.7% to 95% [172].

The estimated burden of this disease has been estimated in 16 countries to date. A total of 21,073,423 cases are estimated for just 16 countries, with majority of these cases in sub-Saharan Africa amongst school children (Table 9). The highest estimated cases were from Nigeria (15,581,400 cases) [62] followed by Kenya with 1,712,676 cases [75], with Senegal, Uganda and Tanzania reporting 1,523,700, 1,300,000 and 420,000 cases respectively [78,122,133]. Algeria is the only North Africa country that has estimated the burden of the disease and they reported 4265 cases [64]. The lowest rates were from Asia, with just 50 and 59 cases estimated in Sri Lanka [90] and Thailand [55] respectively. In between were reports from Denmark (185 cases) [58], Czech republic (960 cases) [68], Austria (1221 cases) [89], Philippines (846 cases) [93]; also reported with slightly higher rates were Uzbekistan (7307 cases) [63], Korea (45,087 cases) [84] and Vietnam with 415,301 [115] estimated cases. Poor socioeconomic status, high population densities, and poor sanitary conditions are some of the factors responsible for the high prevalence of tinea capitis in LMICs.

## 3. Conclusions

In this article, we have attempted to outline the rationale for how the estimates of several serious fungal diseases were made, and the intrinsic limitations to the methodologies used. The term ‘burden’ encompasses annual incidence, period or total prevalence and in the case of RVVC, annual prevalence. During the formulation of each country’s estimate, the authors identified all the pertinent literature and also the gaps. The estimates were not intended as a substitute for high quality epidemiological study or comprehensive surveillance, but do provide a rough approximation of the size of each fungal disease by country and therefore a means of comparing countries. The diagnostic gap between estimated burden and recorded cases numbers provides a clear cut target to close, to improve patient outcomes. The recognition of fungal infections as a major contributor to mortality of several conditions emphasizes the need for public health efforts in reducing the incidence and mortality of these infectious diseases.

## Figures and Tables

**Table 1:** Burden of fungal diseases.

Fungal Disease	Annual Incidence	Global Burden	Comments
**Superficial**			
Skin, hair, nail		~1,000,000,000	
Fungal keratitis		~1,000,000	
**Mucosal**			
Oral candidiasis	~2,000,000		HIV only, 90% of those not on ARVs
Oesophageal candidiasis	~1,300,000		HIV only, 20% on those with CD4 counts <200 and 5% of those on ARVs
Vulvovaginal candidiasis episode			70% affected in their lifetime
Recurrent vulvovaginal candidiasis		~134,000,000	Annual prevalence. Nearly 500 million lifetime experience
**Allergic**			
Allergic bronchopulmonary aspergillosis in asthma		~4,800,000	Adults only, rare in children
Allergic bronchopulmonary aspergillosis in cystic fibrosis		~6675	Adults only, starts from age 4
Severe asthma with fungal sensitisation		~6,500,000	Adults only, probably uncommon in children
Fungal rhinosinusitis		~12,000,000	
**Chronic severe**			
Chronic pulmonary aspergillosis		~3,000,000	
Mycetoma		~9000	1950–2013 case reports, NTD
Chromoblastomycosis		>10,000	Limited data and uncommon, NTD
Coccidioidomycosis		~25,000	
Paracoccidioidmycosis		~4000	
Blastomycosis		~3000	
*Histoplasma* infection	~500,000	~25,000	Most of the new infections are asymptomatic based on skin testing
Sporotrichosis	>40,000		Very limited global data. Very common in hyper endemic regions of Peru, Brazil and Mexico
**Acute invasive**			
Invasive candidiasis	~750,000		Includes 60,000–100,000 cases of intra-abdominal candidiasis
Invasive aspergillosis	>300,000		From about 10 million at risk annually
*Pneumocystis jirovecii* pneumonia in AIDS and non-AIDS	~500,000		
Cryptococcosis in AIDS	~223,000		HIV-related, up to another 10% non-HIV
Mucormycosis	>10,000		Based on French data = 4200.
			Based on Indian data = 910,000
Disseminated histoplasmosis	~100,000		No reliable estimates
Talaromycosis *	~8000		SE Asia only;

* *Talaromyces* (formerly *Penicillium*) *marneffei* infection Data from Brown et al. [1], Vos et al. [10], Armstead et al. [11], Rajasingham et al. [8], Fungal Infection Trust [12], Global Action Fund for Fungal Infection (GAFFI) Roadmap [9], and van de Sande [13]. NTD = WHO-accepted Neglected Tropical Disease.

**Table 2:** Country estimated burden of candidaemia.

Country (Reference)	Burden	Rate/100,000	Comments
Brazil [52]	28,991	14.9	No local incidence data
Pakistan [53]	38,795	21	1.6 per 100,000 + 50% of candida peritonitis
Qatar [54]	288	15.4	15.4 per 100,000 from previous studies
Thailand [55]	8650	13.3	94% of non-neutropenic patients with fungaemia + 4.5% of neutropenic patients
Hungary [56]	1110	11	3–10 per 100,000 from previous studies + 780 cases in chronic ambulatory peritoneal dialysis
Israel [57]	664	11	Incidence rate obtained from a nationwide surveillance between 2005 and 2007 and adjusted to the number of ICU hospital days in 2012
Denmark [58]	527	9.4	Data from Arendrup et al. [30]
Russia [59]	11,840	8.3	0.37 per 1000 hospitalised patients based on previous report
Spain [60]	3807	8.1	8.1 per 100,000 from previous study
United Kingdom [19]	5142	8.1	38% of probable or proven invasive candidiasis tested by blood culture techniques in England, wales and northern Ireland, plus 4.8 cases per 100,000 in Scotland
Ireland [61]	403	6.3	Statistics and Research records office voluntary laboratory reporting programme
Nigeria [62]	9284	6	6 per 100,000 + 50% of candida peritonitis
Uzbekistan [63]	1825	5.9	Data from Ministry of Health
Algeria [64]	2020	5	No local incidence data
Bangladesh [65]	8100	5	No local incidence data
Belgium [66]	555	5	No local incidence data
Chile [67]	878	5	No local incidence data
Czech Republic [68]	526	5	No local incidence data
Dominican Republic [69]	504	5	No local incidence data
Ecuador [70]	1037	5	No local incidence data
Egypt [71]	4127	5	5 per 100,000 and 0.98 per 100,000 of intra-abdominal peritonitis
Greece [72]	541	5	No local incidence data
Guatemala [73]	772	5	No local incidence data
Jamaica [74]	136	5	5.5 per 100,000
Kenya [75]	1990	5	No local incidence data
Mexico [76]	5617	5	5 per 100,000 + 285 in candida peritonitis
Peru [77]	1557	5	2–11 per 100,000 + 50% of candida peritonitis
Tanzania [78]	2181	5	No local incidence data
Trinidad and Tobago [79]	87	5	5 per 100,000 + 285 in candida peritonitis
Ukraine [80]	752	5	5 per 100,000 + 285 in candida peritonitis
Vietnam [81]	4540	5	No local incidence data
Germany [82]	3712	4.6	Data from Statistisches Bundesamt Wiesbaden 2015 [83]
Korea [84]	1976	4.1	0.22 per 1000 patients
France [85]	2370	3.6	No local incidence data
Canada [86]	1034	2.9	Average from 2 studies, 2.8 cases/100,000 [87] and 3.3 cases/100,000 [88] over a 5 year period
Austria [89]	209	2.6	-
Sri Lanka [90]	507	2.5	unpublished data from the department of mycology
Portugal [91]	231	2.2	Data from Portuguese multicentre survey [92]
Philippines [93]	1968	2	2 per 100,000 + 50% candida peritonitis

**Table 3:** Published country estimates of invasive aspergillosis.

Country (Reference)	Burden	Rate/100,000	Assumptions
Vietnam [120]	14,523	16.0	10% of, A.M.L.; 10% of non-AML, 0.5% of renal, T.X.; 4% of lung and liver Tx, 6% of heart Tx, 3.9% of COPD admissions
Egypt [71]	9001	10.7	2.6% of COPD admissions, 10% of, A.M.L.; 10% of all other predisposing haematological malignancies, 1% of renal Tx, 4% of liver Tx, and 2.6% of lung Tx
Greece [72]	1125	10.4	10% of, A.M.L.; 8% of HSCT, 6% of heart Tx, 4% of lung and liver Tx, 1% of kidney, 1.3% of COPD admissions
Algeria [64]	2865	7.1	1.3% of COPD admissions and 2.6% lung cancer, 7.7% neutropenic patients
Ireland [61]	445	7.0	10% of, A.M.L.; 8% of HSCT, 0.5% of renal Tx, 2% of heart Tx, 0.9% of liver Tx, 9.1% of lung Tx, 1.3% of COPD admissions
Israel [57]	574	6.8	10% of, A.M.L.; 10% of HSCT, 0.5% of renal Tx, 4% of lung, T.X.; 6% of heart Tx, 4% of liver Tx, 1.3% of COPD admissions
Belgium [66]	675	6.1	10% of, A.M.L.; 0.5% of renal Tx 4% of lung and liver Tx, 6% of heart Tx, 1.3% of COPD admissions
Pakistan [53]	10,949	5.9	3.9% of COPD admissions, 2.6% of lung cancer Tx, 10% of, A.M.L.; 10% of non-AML
Ecuador [70]	748	5.5	10% of, A.M.L.; 8% of HSCT, 1% of Liver Tx, 0.5% renal of Tx, 2% of heart Tx, 9% of lung Tx, 1.3% of COPD admissions
Denmark [58]	294	5.3	10% of HSCT, 6% of heart and lung Tx, 4% of liver, T.X.; 1% in renal, T.X.; 1.3% of COPD admission
Bangladesh [65]	5166	5.1	1.3% of COPD (only COPD included in the assumptions)
Germany [82]	4280	5.1	5% of, A.M.L.; 5% of non-AML haematological malignancies, 1% of renal Tx, 20% of lung Tx, 6% of heart Tx, 4% of liver Tx and 1.3% of COPD admissions
Peru [77]	1621	5.0	10% of, A.M.L.; 10% of non-AML haematological malignancies, 1.3% of COPD admissions, 0.3% of renal Tx, 0.8% of liver Tx, 4.8% of heart Tx, 42% of HSCT
Uzbekistan [63]	1521	4.8	50% of, A.M.L.; 1.3% of COPD admissions
United Kingdom [19]	2912	4.6	9% of HSCT, 10% of, S.O.T.; 0.6% of, H.I.V.; 15% in haematological disease, 1.3% of COPD admissions
Brazil [52]	1854	4.5	13.4% of, A.M.L.; 2.3% of HSCT receipients, 0.5% of renal Tx, 13.3% of lung Tx (no COPD data)
Korea [84]	2150	4.5	14.7% of HSCT, 0.76% of liver Tx, 0.24% of renal Tx, 8.8% of lung Tx, 0.8% of heart Tx, and 5% of AML or non-AML haematological malignancy, 1.6% of COPD admissions
Guatemala [73]	671	4.3	10% of, A.M.L.; 10% in Non-AML haematological malignancies, 1.3% of COPD admissions
Austria [89]	333	4.1	-
Mexico [76]	4510	4.0	1.3% of COPD admissions, 8% of haematological conditions, 0.7% of solid organ Tx
Nepal [121]	1119	4.0	3.9% of COPD admissions, 10% of, A.M.L.; 10% of non-AML haematological malignancies
Uganda [122]	389	3.8	7% in haematological malignancy, 1.3% of COPD admissions
Hungary [56]	319	3.2	12% of, A.M.L.; 6–8 % of HSCT, 8.6% of lung Tx, 3.4% of heart Tx, 4.7% of Liver Tx, 1.3% of renal Tx, 1.35% of COPD admissions
Philippines [93]	3085	3.0	10% of, A.M.L.; 10% of non-AML, 0.5% of renal Tx, 4% of liver Tx, 1.3% of COPD admissions
Czech Republic [68]	297	2.8	1.3% of COPD admissions, 10% of, A.M.L.; 1% of renal Tx, 5% of lung Tx, 5.5% of heart Tx, 4% in liver Tx
Spain [60]	1293	2.8	10% of HSCT, 6% of heart Tx, 4% of lung and liver Tx, 1% of kidney Tx, 7% of, A.M.L.; 7.7% of non-AML haematological disease, 1.3% of admitted COPD cases
Ukraine [80]	1233	2.7	10% of, A.M.L.; 10% of non-AML, 3.6% of COPD admissions
Portugal [91]	243	2.3	1.3% of COPD admissions, 2.63% of lung cancers, 31% of HSCT, 6% of heart Tx, 4% of lung and liver Tx, 1% of kidney Tx
Russia [59]	3238	2.3	10% of, A.M.L.; 20% of HSCT, 1% of renal Tx, 4% of liver Tx, 6% of heart Tx, 1.3% of admitted COPD patients
France [85]	1185	1.8	1.8 per100,000 from previous studies and 1300 cases a year in COPD.
Chile [67]	296	1.7	10% of Leukaemia, 1.3% of COPD admission, 2.6% of lung cancers
Canada [86]	566	1.6	8.9% of, A.M.L.; 0.2% of kidney Tx, 0.5% of liver, 3.8% of lung Tx, 0.8% of heart Tx and 0.2% in pancreas Tx, and 7.5% in HSCT, 3.6/1000 COPD admissions
Thailand [55]	941	1.4	13.5% of, A.M.L.; 3% of renal Tx, 4% of lung and liver Tx, 1.3% of COPD
Sri Lanka [90]	229	1.1	10% of, A.M.L.; 100% of non-AML, 0.5% of renal Tx, 4% of liver Tx
Dominican Republic [69]	61	0.8	10% of, A.M.L.; 10% of non-AML haematological patients, 13.4% of COPD over 40 years
Kenya [75]	239	0.6	10% of, A.M.L.; 10% of non-AML haematological patients (COPD was not included)
Nigeria [62]	928	0.6	10% of, A.M.L.; 10% of non-AML haematological malignancies (COPD was not includesd)
Qatar [54]	11	0.6	11 cases recorded in the reference laboratory
Trinidad and Tobago [79]	8	0.6	10% of AML (COPD population was not included)
Tanzania [78]	20	0.1	10% of AML (COPD population was not included)

AML: Acute myeloid leukaemia. COPD: Chronic obstructive pulmonary disorder. HSCT: Haematopoietic stem cell transplant. Tx: Transplant. SOT: Solid Organ Transplant.

**Table 4:** Estimated burden of *Pneumocystitis jirovecii* pneumonia.

Country (Reference)	Burden	Rate/100,000	Assumptions
Nigeria [62]	74,595	48.2	40% of new AIDS cases in children and 10% in adults
Kenya [75]	17,000	43.0	10% in HIV with CD4 < 200
Trinidad and Tobago [79]	400	30.0	80% of HIV patients with CD < 200
Tanzania [78]	9600	22.0	10.4% of adults living with HIV
Ukraine [80]	6152	13.5	60% of HIV patients
Jamaica [74]	350	13.0	255 in HIV HAART naïve
Senegal [133]	1149	8.2	22% of new AIDS
Uzbekistan [63]	165	5.37	60% of HIV with CD < 200
Guatemala [73]	722	4.7	4.7% in HIV patients
Peru [77]	1447	4.6	13% in AIDS
Mexico [76]	5130	4.5	24% in HIV
Chile [67]	766	4.3	35% of new HIV cases
Nepal [121]	990	3.6	16.7% of new AIDS case in children and 22.4% of adults
Spain [60]	305	3.4	3.4/100,000 from previously published studies
Ecuador [70]	535	3.28	10.7% of HIV with CD4 < 200 from previous study
Thailand [55]	1708	2.6	21% of new AIDS cases
Dominican Republic [69]	234	2.31	80% of HIV HAART naïve partients with CD4 < 200
Brazil [52]	4115	2.1	4.7% of AIDS patients with CD4 < 350
Germany [82]	1013	1.3	Epidemiological data from Robert Koch institute 2012
Pakistan [53]	2200	1.2	16% in HIV
Belgium [66]	120	1.1	From unpublished data from the an AIDS reference from Leuven, Belgium
France [85]	658	1.0	Data from retrospective study in 2014
Greece [72]	112	1.0	26.2% of the new AIDS cases
Uganda [122]	412	1.0	2.6% of non-HIV pneumonia deaths in children below 15 years + 36.8% of HIV adults HIV patients with CD4 < 100 + 10–49% of pneumonia admissions in children with HIV
Ireland [61]	50	0.8	25% in HIV
Qatar [54]	15	0.8	15 cases reported to the reference laboratory
Canada [86]	252	0.71	Cases from a single tertiary centre in Montreal
Czech Republic [68]	72	0.7	Internal registry of the Department of tropical and infectious diseases in Prague
Vietnam [120]	608	0.67	13% of new AIDS diagnosis
Portugal [91]	65	0.62	0.61/100,000 on a previous study
Korea [84]	245	0.51	20% of new HIV patients with low CD4
Philippines [93]	391	0.4	31% of new AIDS diagnosis
United Kingdom [19]	207	0.33	Incidence rates in HIV and solid organ transplant recipients from previous studies.
Israel [57]	26	0.3	Records from Clinical Microbiological lab at Tel Aviv
Algeria [64]	74	0.18	15% in HIV CD4 < 200 from various studies
Russia [59]	1414	0.16	2.1% of new HIV
Egypt [71]	125	0.15	1.9% of HIV based on previous study
Hungary [56]	5	0.1	Records from clinical laboratory
Bangladesh [65]	58	0.04	17% of HIV
Denmark [58]	2	0.04	35% in new AIDS patient from data of 1997–2009 and 255 in Non-AIDS based on previous study

**CD**: Cluster of differentiation. **HAART**: Highly active anti-retroviral therapy.

**Table 5:** Country estimates of the burden of chronic pulmonary aspergillosis.

Country (Reference)	Burden	Rate/100,000
Russia [59]	52,311	126.2
Nigeria [62]	120,747	78
Philippines [93]	77,172	78
Pakistan [53]	72,438	70
Vietnam [120]	55,509	61
Dominican Republic [69]	1374	55
Uganda [122]	18,000	46
Bangladesh [65]	20,720	41
Kenya [75]	12,927	32
Thailand [55]	19,044	29.2
Belgium [66]	662	27.7
Quatar [54]	176	26.8
Nepal [121]	6611	24.2
India [136]	209,147	24
Tanzania [78]	10,437	24
Korea [84]	10,754	22.4
Ukranie [80]	10,054	22
Senegal [133]	2700	19
Mexico [76]	18,246	15.9
Sri Lanka [90]	2886	14.4
Egypt [71]	3015	13.8
Peru [77]	3593	11
Guatemala [73]	1484	9.6
Spain [60]	4318	9.19
Trinidad and Tobago [79]	110	8.2
Chile [67]	1212	6.9
Uzbekistan [63]	1941	6.3
Brazil [52]	12,032	6.2
Hungary [56]	504	6
United Kingdom [19]	3600	5.7
France [85]	3450	5.2
Denmark [58]	270	4.8
Austria [89]	328	4.7
Greece [72]	386	3.7
Czech Republic [68]	365	3.5
Ecuador [70]	2100	3.28
Ireland [61]	196	3.1
Portugal [91]	776	3.1
Jamaica [74]	82	3
Germany [82]	2320	2.9
Israel [57]	200	2.5
Algeria [64]	897	2.2
Canada [86]	492	1.4

**TB**: Tuberculosis. **NTM**: Non-tuberculous mycobacteria.

**Table 6:** Estimated burden of allergic bronchopulmonary aspergillosis.

Country (Reference)	Burden	Rate/100,000	Comments
United Kingdom [19]	235,070	372	2.5% of asthma + 12.5% of adult CF and 7.5% of children with CF
Trinidad and Tobago [79]	3491	260	2.5%of asthma
Dominican Republic [69]	25,149	249	2.5% of asthma
Belgium [66]	23,119	208.3	2.5% of asthma patients and 15% in CF
Brazil [52]	390,486	201.3	2.1% adult asthmatics and 22% adult with CF
Greece [72]	20,843	193	2.5% of asthma and 17.7% of CF
Jamaica [74]	5116	188	2.5% of asthma
Ecuador [70]	26,642	185	2.5% of asthma
Canada [86]	61,854	174	2.5% in Asthma and 18% in CF
Egypt [71]	133,834	162	2.5% of asthma
Spain [60]	59,210	156	2.5% of asthma
Germany [82]	123,960	154	2.5% of asthma and 4.8% of CF
France [85]	95,331	145	2.5% of asthma
Ireland [61]	8960	140	2.5% of asthma and 17.75% in CF
Hungary [56]	13129	132.5	2.5%of asthma and 15% in CF
Denmark [58]	7328	131	2.5% of asthma and 5% CF
Philippines [93]	121,113	123	2.5% of asthma
Russia [59]	175,082	122.5	2.5% of asthma
Portugal [91]	12,600	119	2.5% of asthma
India [136]	1,380,000	114	2.5% (0.7–3.5%) of asthma
Israel [57]	8297	101	2.5% of asthma and 6.6% in CF
Chile [67]	17,183	97.9	2.5% of asthma
Austria [89]	7537	91.7	-
Algeria [64]	31,310	77	2.5% of asthma patients
Peru [77]	22,453	72	2.5% of asthma
Senegal [133]	9976	71	2.5% of asthma
Ukranie [80]	28,447	62.4	2.5%of asthma
Nigeria [62]	93,649	60.5	2.5% of asthma
Qatar [54]	1126	60.2	2.5% of asthma
Mexico [76]	47,855	60	2.5% of asthma
Thailand [55]	38,009	58.4	2.5% of asthma
Korea [84]	27,312	56.9	2.5% of asthma
Bangladesh [123]	90,262	56	2.5% of asthma patients
Pakistan [53]	94,358	51	2.5% of asthma
Sri Lanka [90]	10,344	49	2.5% of asthma
Uganda [122]	18,700	47.9	2.5% of asthma
Czech Republic [68]	4739	45	2.5% of asthma and 18% CF
Kenya [75]	17,696	44	2.5% of asthma
Tanzania [78]	18,987	44	2.5% of asthma
Guatemala [73]	5568	36.1	2.5%of asthma
Nepal [121]	9546	35	2.5% of asthma
Vietnam [120]	23,607	23	2.5% of asthma
Uzbekistan [63]	879	2.9	2.5% of asthma + 15% of CF

CF: Cystic fibrosis.

**Table 7:** Estimated burden of severe asthma with fungal sensitisation.

Country (Reference)	Burden	Rate/100,000
United Kingdom [19]	413,724	654
Trinidad and Tobago [79]	4608	344
Dominican Republic [69]	33,197	329
Ecuador [70]	45,183	311
Brazil [52]	599,283	288
Belgium [66]	30,402	273
Greece [72]	27,744	256
Jamaica [74]	6753	248
Egypt [71]	176,661	214
Canada [86]	73,344	206
Germany [82]	163,131	203
Spain [60]	93,044	198
France [85]	124,678	189
Ireland [61]	111,675	182
Hungary [56]	17,330	175
Philippines [93]	159,869	162
Russia [59]	231,000	161
Portugal [91]	16,614	159
Denmark [58]	7793	139
Chile [67]	22,300	127
Austria [89]	9949	121
Algeria [64]	41,329	102
Peru [77]	29,638	95
Senegal [133]	13,168	93
Ukraine [80]	37,491	82
India [136]	960,000	80
Qatar [54]	1486	80
Nigeria [62]	120,753	78
Thailand [55]	50,172	77
Korea [84]	36,052	75
Bangladesh [65]	119,146	74
Pakistan [53]	129,776	70
Israel [57]	5540	68
Sri Lanka [90]	13,654	65
Czech Republic [68]	6581	62
Uganda [122]	24,684	62
Kenya [75]	23,359	58
Tanzania [78]	25,063	57
Mexico [76]	66,997	53
Guatemala [73]	7349	48
Nepal [121]	12,600	46
Vietnam [120]	31,161	34
Uzbekistan [63]	1147	3.7

**Table 8:** Country estimates of the burden of fungal keratitis.

Country	Burden	Rate/100,000	Proportion of Microbial Keratitis that is Fungal
Nepal [121]	19,938	73.00	27–62% of microbial keratitis
Pakistan [53]	80,553	44.00	0.15% of general population based on the Chinese study [150]
Thailand [55]	9765	15.00	15% of microbial keratitis
Egypt [71]	11,550	14.00	40% of microbial keratitis
Mexico [76]	11,638	10.40	0.15% of general population based on the Chinese study [150]
Vietnam [120]	6356	7.00	Based on previous Vietnamese study reporting a rate of 7 per 100,000 [160]
Sri Lanka [90]	100,000	6.30	40% direct microscopy positivity rate for fungal elements in corneal buttons and scrapings
Qatar [54]	6	1.68	6 cases recorded in the mycology reference laboratory
China [161]	17,038	1.30	0.15% of general population [150]
Philippines [93]	358	0.36	Based on cases seen at a tertiary government hospital in the national capital region in 2015
Korea [84]	29	0.06	Based on a previous Danish study [162]
Denmark [58]	3	0.05	Based on a previous Danish study [162]
Germany [82]	32	0.04	Based on a previous Danish study [162]

**Table 9:** Prevalence of Tinea capitis among school-age children in sub-Saharan Africa.

Prevalence (%)	Country	Year of Publication	Reference
76.1	Nigeria	2011	Adefemi et al. [169]
68.0	Kenya	2015	Moto et al. [175]
49.5	Rwanda	1983	Buginco et al. [176]
45.0	Nigeria	2016	Dogo et al. [171]
44.8	Senegal	2016	Diongue et al. [177]
39.3	Mali	2016	Coulibaly et al. [178]
36.5	Ethiopia	2015	Leiva-Salinas et al. [179]
35.2	Nigeria	2015	Kalu et al. [180]
33.3	Kenya	2001	Ayaya et al. [181]
31.2	Nigeria	2008	Ayanbimpe et al. [182]
26.9	Nigeria	2014	Oke et al. [170]
23.1	Gabon	2011	Hogewoning et al. [183],
23.1	Gabon	2013	Hogewoning et al. [184]
22.5	Tanzania	1998	Frederick et al. [185]
20.6	Kenya	2013	Hogewoning et al. [184]
15.4	Nigeria	2014	Ayanlowo [186]
13.9	Ivory Coast	2013	Fulgence et al. [187]
11.2	Kenya	2009	Chepchirchir et al. [188]
9.4	Nigeria	2008	Emele et al. [189]
8.7	Ghana	2013	Hogewoning et al. [184]
8.4	Ghana	2013	Hogewoning et al. [184]
8.1	Cameroon	2014	Kechia et al. [190]
7.8	Kenya	1997	Schmeller et al. [191]
7.1	Kenya	2010	Komba et al. [192]
3.6–9.6	Mozambique	2007	Sidat et al. [193]

## Supplementary Materials

Supplementary File 1
