# Supplementary material for: Global and Multi-National Prevalence of Fungal Diseases—Estimate Precision
Source: J Fungi (Basel). 2017 Oct 18;3(4):57. doi: 10.3390/jof3040057 (PMC5753159; doi:10.3390/jof3040057)
Supplement: Supplementary File 1 [file jof-03-00057-s001.pdf]

Fungal diseases kill more than 1.5 million and affect over a billion people. However, they are still a neglected topic by public health authorities even though most deaths from fungal diseases are avoidable. Serious fungal infections occur as a consequence of other health problems including asthma, AIDS, cancer, organ transplantation and corticosteroid therapies. Early accurate diagnosis allows prompt antifungal therapy; however this is often delayed or unavailable leading to death, serious chronic illness or blindness. Recent global estimates have found 3,000,000 cases of chronic pulmonary aspergillosis, ~223,100 cases of cryptococcal meningitis complicating HIV/AIDS, ~700,000 cases of invasive candidiasis, ~500,000 cases of *Pneumocystis jirovecii* pneumonia, ~250,000 cases of invasive aspergillosis, ~100,000 cases of disseminated histoplasmosis, over 10,000,000 cases of fungal asthma and ~1,000,000 cases of fungal keratitis occur annually (**Table 1**). Since 2013, the Leading International Fungal Education (LIFE) portal has facilitated the estimation of the burden of serious fungal infections country by country for over 5.7 billion people (>80% of the world's population) (**Figure 1**). The estimates were not intended as a substitute for high quality epidemiological study or comprehensive surveillance, but do provide a rough approximation of the size of each fungal disease by country and therefore a means of comparing countries. The diagnostic gap between estimated burden and recorded cases numbers provides a clear cut target to close, to improve patient outcomes. The recognition of fungal infections as a major contributor to mortality of several conditions emphasizes the need for public health efforts in reducing the incidence and mortality of these infectious diseases.

Fungal Disease	Annual Incidence	Global Burden
**Superficial**		
Skin, hair, nail		~1,000,000,000
Fungal keratitis		~1,000,000
**Mucosal**		
Oral candidiasis	~2,000,000	
Oesophageal candidiasis	~1,300,000	
Vulvovaginal candidiasis episode	70% affected in their lifetime	
Recurrent vulvovaginal candidiasis		~134,000,000
**Allergic**		
ABPA in asthma		~4,800,000
ABPA in cystic fibrosis		~6,675
Severe asthma with fungal sensitisation		~6,500,000
Fungal rhinosinusitis		~12,000,000
**Chronic severe**		
Chronic pulmonary aspergillosis		~3,000,000
Mycetoma		~9,000
Chromoblastomycosis		>10,000
Coccidioidomycosis		~25,000
Paracoccidioidmycosis		~4,000
Blastomycosis		~3,000
Histoplasma infection	~500,000	~25,000
Sporotrichosis	>40,000	
**Acute invasive**		
Invasive candidiasis	~750,000	
Invasive aspergillosis	>300,000	
PCP in AIDS and non-AIDS	~500,000	
Cryptococcosis in AIDS	~223,000	
Mucormycosis	>10,000	
Disseminated histoplasmosis	~100,000	
Talaromycosis	~8,000	

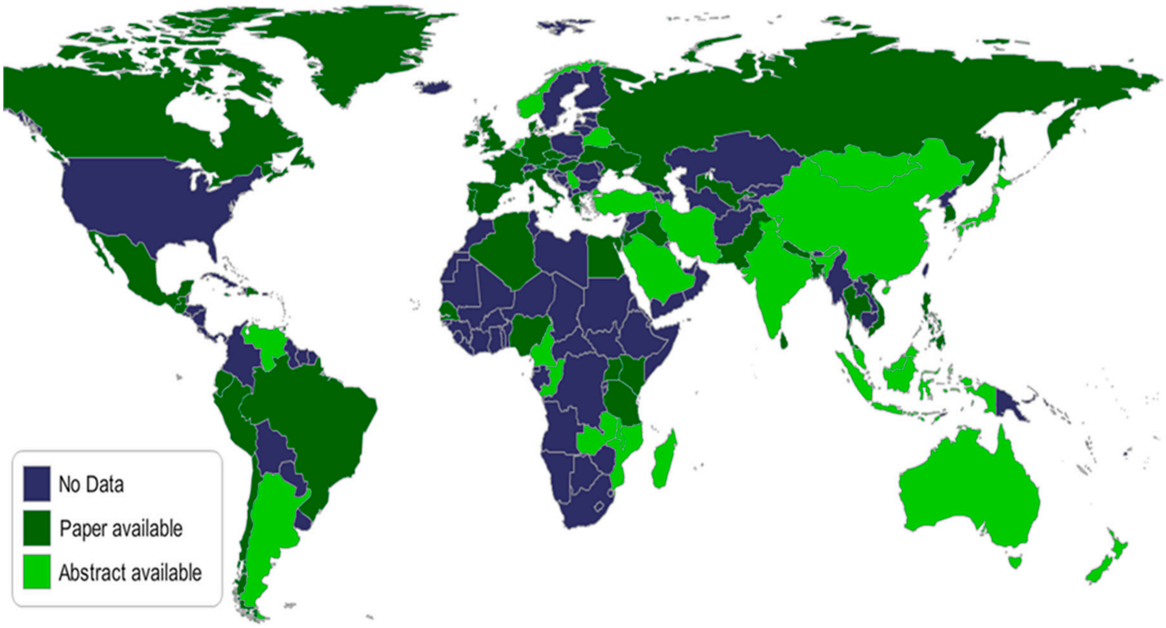
No Data
Paper available
Abstract available
